# Sphingolipids Are Required for Efficient Triacylglycerol Loss in Conjugated Linoleic Acid Treated Adipocytes

**DOI:** 10.1371/journal.pone.0119005

**Published:** 2015-04-23

**Authors:** Wei Wang, Michael Fromm

**Affiliations:** 1 Department of Animal Science, University of Nebraska, Lincoln, Nebraska, United States of America; 2 Center for Biotechnology, University of Nebraska, Lincoln, Nebraska, United States of America; Faculty of Biology, SPAIN

## Abstract

Conjugated linoleic acid (CLA) reduces adiposity in human and mouse adipocytes. This outcome is achieved through a variety of biological responses including increased energy expenditure and fatty acid oxidation, increased inflammation, repression of fatty acid biosynthesis, attenuated glucose transport, and apoptosis. In the current study, profiling of 261 metabolites was conducted to gain new insights into the biological pathways responding to CLA in 3T3-L1 adipocytes. Sphinganine and sphingosine levels were observed to be highly elevated in CLA treated adipocytes. Exogenous chemicals that increased endogenous ceramide levels decreased lipid levels in adipocytes, and activated AMP-activated protein kinase (AMPK) as well as NF-κB, both of which are typically activated in CLA treated adipocytes. Concurrent inhibition of ceramide *de novo* biosynthesis and recycling from existing sphingolipid pools attenuated the lipid lowering effect normally associated with responses to CLA, implicating ceramides as an important component of the lipid lowering response in CLA treated adipocytes.

## Introduction

The *trans*-10, *cis*-12 isomer of CLA (*t*10*c*12 CLA) is capable of reducing adiposity in human and mouse adipocytes [1–5]. Molecular responses to *t*10*c*12 CLA include activation of AMP-activated protein kinase (AMPK) [6], sirtuin 1 [7], and attenuation of peroxisome proliferator-activated receptor γ (PPARγ) protein levels [3]. Treatment with *t*10*c*12 CLA requires nuclear factor-kappa B (NF-κB) for an inflammatory response [3,4,8–11] that includes increased prostaglandin biosynthesis in human adipocytes [12], in mouse white adipose tissue [13], and in 3T3-L1 adipocytes [14]. Despite this progress in understanding the pathways involved in the early perception of *t*10*c*12 CLA and the complex regulation of the subsequent response, much remains unknown in this process.

Sphingolipids are a complex class of lipids containing the sphingoid backbones of sphinganine (dihydrosphingosine) and sphingosine. Attaching sphinganine or sphingosine to a fatty acid via an amide linkage forms ceramide, the simplest sphingolipid that is the starting point for more complex sphingomyelins, cerebrosides, and gangliosides [15]. Sphingolipids have structural roles in lipid membranes and can serve as sources of signaling molecules as well. Signaling pathways affected by sphingolipids or molecules derived from them, include apoptosis, inflammation, mitochondrial function, and general cell metabolism [16,17].

The role of sphingolipids in adipocytes and obesity is still emerging. A study of genetically obese mice (*ob/ob*) and high-fat diet-induced obese mice found inhibiting ceramide *de novo* synthesis attenuated obesity symptoms, facilitating weight reduction, better energy metabolism, and improved insulin sensitivity [18]. In 3T3-L1 adipocytes, ceramide levels decreased as pre-adipocytes differentiated into adipocytes, and increased when adipocytes were treated with epigallocatechin gallate, which reduced adipocyte fat content [19].

In this study we performed metabolic profiling of *t*10*c*12 CLA treated adipocytes to gain insight into responding pathways. Our analysis of adipocytes after 12 h of exposure to *t*10*c*12 CLA indicated that in addition to the expected changes in lipid pathways, there were significantly increased sphinganine and sphingosine levels in the ceramide pathway. This study focused on the ceramide pathway due to its important roles in many aspects of cell biology and cell signaling [15–17]. Ceramide levels were experimentally perturbed to determine ceramide’s functional contribution to lowering triacylglycerol in *t*10*c*12 CLA treated adipocytes.

## Materials and Methods

### Reagents

Insulin (I1882), isobutyl-methylxanthine (I5879), dexamethasone (D4902), bovine serum (A2153), calf serum (12138C), N-acetyl-D-sphingosine (C2 ceramide, A7191), monoclonal anti-ceramide antibody (C8104), myriocin (M1177), fumonisin B1(F1147) and SKI-II (S5696) were from Sigma (St. Louis, MO). AMPK inhibitor compound C (17126) was from Calbiochem (San Diego, CA). Antibodies to β-actin (SC-30656), NF-κB p65 (sc-372), sphingosine-1-phosphate phosphatase 1(Sppase, sc-55306) and serine palmitoyltransferase (Sptlc, sc-136076) were from Santa Cruz Biotechnology (Santa Cruz, CA). Antibodies to phospho-AMPKα pThr172 (2531) and AMPK (2532) were purchased from Cell Signaling (Beverly, MA). T7 RNA polymerase (P2075), RNase-Free DNaseI (M6101) and rNTPs (E6000) were purchased from Promega (Madison, WI). DharmaFECT Duo transfection reagent (T2010) was from Dharmacon (Thermo Fisher Scientific, Boulder, CO). Chemicals were dissolved in DMSO, with the exception that C2-ceramide was dissolved in ethanol, and fumonisin B1 was dissolved in water. The lowest effective chemical concentrations were chosen by testing a range of initial concentration and were as follows: 20 μmol/L fumonisin B1, 50 μmol/L myriocin, 10 μg/mL SKI-II, 30 μmol/L C2-ceramide, and were added 1 h before adding fatty acids. Chemical stock solutions were added directly to media at ≤0.2% of the final volume of the media.

### 3T3-L1 cell culture and differentiation

3T3-L1 fibroblasts [20] were cultured in Dulbecco's modified Eagle's medium (DMEM; Invitrogen, Carlsbad, CA) containing 10% bovine calf serum (Fisher, Pittsburgh, PA). Cells were seeded at a concentration of 1 x 10^4^ cells/cm^2^ and differentiated as described [7], reaching a density of ~4.5 x 10^4^ cells/cm^2^. The percentage of adipocytes was predominantly 90% of the cells after differentiation, although higher passage cells occasionally produced only 60% of the cells as adipocytes after differentiation. Our earlier whole genome microarray analyses of non-differentiated pre-adipocytes indicated they demonstrate very small responses to *t*10*c*12 CLA [14], suggesting the majority of the cellular responses to *t*10*c*12 CLA in a mixed population after differentiation are from adipocytes.

### Fatty acid preparation

Fatty acids (>99%, Nu-check Prep, Elysian, MN), either linoleic acid (LA) or trans-10, cis-12 CLA, were dissolved in 0.1 M KOH, diluted into fatty acid free (>99%) bovine serum albumin (BSA) in phosphate buffered saline at a 1:1 ratio (2 mmol/L BSA: 2 mmol/L fatty acid), pH adjusted to 7.4, and added to the cultures containing 4 to 6 d post-differentiated 3T3-L1 adipocytes [7]. All media contained 100,000 U/L penicillin and 172 μmol/L streptomycin (Invitrogen, Carlsbad, CA). 50 μM *t*10*c*12 CLA was used if assaying chemicals that increased triacylglycerol loss as this facilitated observing triacylglycerol loss, but otherwise 100 μM *t*10*c*12 CLA was generally used.

### Metabolic profiling

Metabolite profiling of 3T3-L1 adipocytes was performed by Metabolon (Durham, NC). Six replicates of 3T3-L1 adipocytes, treated with 100 μM LA or *t*10*c*12 CLA for 0.5 h or 12 h, were collected, and frozen at -80°C for later analysis. Samples were solvent extracted and analyzed by GC/MS and LC/MS or LC/MS/MS platforms for 261 metabolites (S1 Dataset). Compounds were identified by known standards or prior characterizations of metabolite libraries. Welch’s two–sample *t*-tests were used to identify biochemicals that differed significantly between LA and *t*10*c*12 CLA treatments.

### RNA interference

Four to five days post differentiation 3T3-L1 adipocytes were transfected by DharmaFECT Duo transfection reagent as described [21]. The final transfection reagent concentration was 1.4 μl/cm^2^. DharmaFECT Duo and 100 nmol/L shRNA were added 24h before adding fatty acids. For drug and shRNA combination assays, drugs were added to media 1h before fatty acid treatments.

### shRNA preparation

Initial sequence against targets were obtained from literature [22,23] or online tools (http://sirna.wi.mit.edu/, http://www.thermoscientificbio.com/design-center/?redirect=true). Five potential sequences for each target were chosen and prepared as described to determine the most effective ones for shRNA knockdowns [24]. In brief, shRNA sequences were synthesized using the T7 polymerase (Promega, Madison, WI) templates and purified by 12–20% denaturing polyacrylamide gel. The shRNA sequences used were: scramble (sh-Non): 5′-AAC AGU CGC GUU UGC GAC UGG UCU CUU GAA CCA GUC GCA AAC GCG ACU GCC UAU AGU GAG UCG UAU UA-3′; ShRNA-sphingosine-1-phosphate phosphatase 1 (sh-S1PP): 5′-AAU CAU CAA GCU GGA GGU CUU CUU CUC UUG AAA GAA GAC CUC CAG CUU GAU GAC CUA UAG UGA GUC GUA UUA-3′; or ShRNA-serine palmitoyltransferase long chain subunit 1 (sh-SPTLC): 5′-AAG CCA UCA UUU ACU CGU AUG UCU CUU GCA CAU ACG AGU AAA UGA UGG CCC UAU AGU GAG UCG UAU UA-3′.

### Western blot

Nuclear and cytosolic extracts were isolated using a nuclear extract kit (Active Motif, Carlsbad, CA). Equal amounts of proteins were separated by SDS-PAGE, transferred to Immun-blot PVDF membrane (Bio-Rad Laboratories, Hercules, CA), probed with the indicated primary antibodies, and detected with secondary antibodies. Enhanced chemiluminescence (Pierce, Rockford, IL) was used for detection. Band intensities were determined from digital images from exposures in the linear range using software (Quantity One, Biorad, Hercules, CA). All western blot analyses were repeated at least three times.

### Ceramide assay

Immunodot blots for ceramide levels were performed as described with modifications [25–27]. Equal amount of cells (~4.5x10^5^) in 6-well plates from each treatment were collected by spinning down at 100 x g for 5 min. Cells were resuspended in 500 μl ice-cold acidic methanol (acetic acid: methanol 1:50). Lipids were extracted by addition of 500 μl chloroform and 500 μl H_2_O. The organic lower-phase was collected and evaporated with nitrogen gas. After adding 100 μl of chloroform/methanol (2:1, v/v) to each tube, 2 μl of each sample was spotted on PVDF membrane and allowed to dry for 30 min. The PVDF membrane was blocked with 5% dry milk in PBS at room temperature for 1 hour, and standard immunoblot procedures followed to detect ceramide levels using a monoclonal antibody that detects ceramides (Sigma C8104 clone MID 15B4, Saint Louis, MO, USA).

### Quantification of cellular triacylglycerol amounts

Cells (~4.5x10^5^) were washed twice with 1 ml PBS, collected in 1 ml PBS by scraping with a rubber policeman spatula, dispersed by pipetting, and aliquoted for triacylglycerol (0.2 ml), DNA (0.1 ml), and immunoblot assays (0.7 ml). A 0.2 ml aliquot of cells was centrifuged at 200 x g for 5 min, the cell pellet was re-suspended in 0.2 ml Triglyceride Working Reagent (TR0100; Sigma, St. Louis, MO) and sonicated for 6s to solubilize the cells and oil bodies. The resulting solution was incubated at 37°C for a 5 min reaction and then cooled. The absorbance at 540 nm was measured and the triacylglycerol level was determined against a standard curve. Values were normalized to the cellular DNA content for the corresponding well. Triacylglycerol data are expressed as nmol of triacylglycerol per μg of DNA. A comparison of this sonication method with a method using isopropanol-hexane extraction [28] prior to the triacylglycerol assay indicated the two methods gave similar linear responses (S1 Fig).

### Quantification of DNA content

The DNA assay was performed using Hoechst 33342 (Sigma B2261) and a standard protocol (http://www.piercenet.com/instructions/2162245.pdf) with the following modifications. Cells were harvested and sonicated as described above in the triacylglycerol assay. A 10μl aliquot of the sonicated lysate was added to 200μl of a 1 μg/ml Hoechst 33342 working solution. The mixed solution was incubated at room temperature in the dark for 10 min and then a 350 nm excitation was used for measuring the fluorescence emission at 460 nm. A standard curve of diluted DNA (Sigma D1626) was used to determine the DNA concentration of the samples.

### Statistical Analysis

One or two-way ANOVA was used to analyze the data. Posthoc pairwise comparisons were calculated using Tukey’s test and were considered significant for *p* ≤ 0.05. All analyses were performed using SAS software (SAS, Cary, NC).

## Results

### Metabolite profiling of *t*10*c*12 CLA treated 3T3-L1 adipocytes

**Fig 1:**
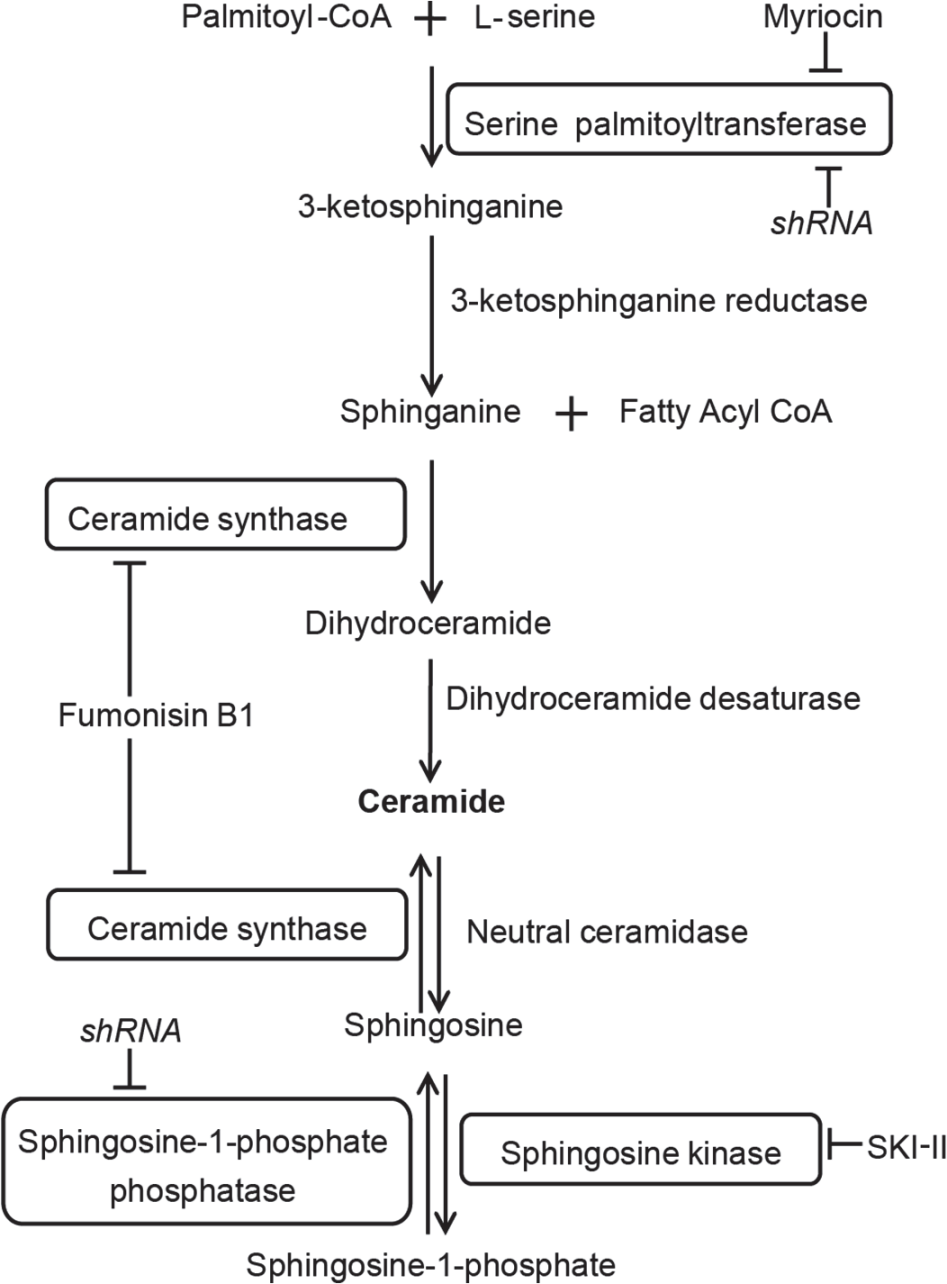
Biosynthetic pathway of ceramide and sphingolipids. Entry into the pathway occurs by biosynthesis via serine palmitoyltransferase or dephosphorylation of sphingosine-1-phosphate by sphingosine-1-phosphate phosphatase. The enzyme targets of myriocin, fumonisin B1, and sphingosine kinase inhibitor SKI-II are also shown as are the targets of shRNA used in this study.

A metabolic profiling analysis of 3T3-L1 adipocytes treated with either 100 μM LA or *t*10*c*12 CLA for 0.5 h or 12 h was conducted for the purpose of identifying early changes in metabolites in these cells. A total of 261 metabolites were surveyed in this analysis (S1 Dataset). At 0.5 h, the main change was the large increase in *t*10*c*12 CLA relative to the LA controls, indicating rapid uptake of *t*10*c*12 CLA by the adipocytes. At 12 h, there were changes in various lipid levels, consistent with the ability of *t*10*c*12 CLA to perturb lipid metabolism (S1 Dataset). The most striking change and focus of the present study was a 9.8 fold increase in sphinganine and a 3.6 fold increase in sphingosine (Table 1). This indicates the sphingolipid pathway is highly elevated 12 h after adding *t*10*c*12 CLA. Sphinganine is produced early in the biosynthetic pathway for ceramides, while sphingosine is derived from ceramide or from recycling of sphingolipids (Fig 1). Ceramide levels were not measured in this metabolite profiling analysis, and were measured separately as described below.

**Table 1:** Relative sphingolipid levels in 3T3-L1 adipocytes after *t*10*c*12 CLA treatment.

^1^Metabolite	CLA vs LA at 0.5 h	CLA vs LA at 12 h
Sphinganine	1.46	9.81*
Sphingosine	1.38	3.65*

^1^Six replicates of 3T3-L1 adipocytes, treated with 100 μM LA or *t*10*c*12 CLA for 0.5 or 12 h, were analyzed by metabolite profiling on GC/MS, LC/MS, and LC/MS/MS systems (Metabolon, Durham, NC).

*p ≤ 0.05, for a Welch’s t-test between the LA and CLA treatments at each time.

### Ceramide levels increase in *t*10*c*12 CLA treated adipocytes and cause triacylglycerol reductions

**Fig 2:**
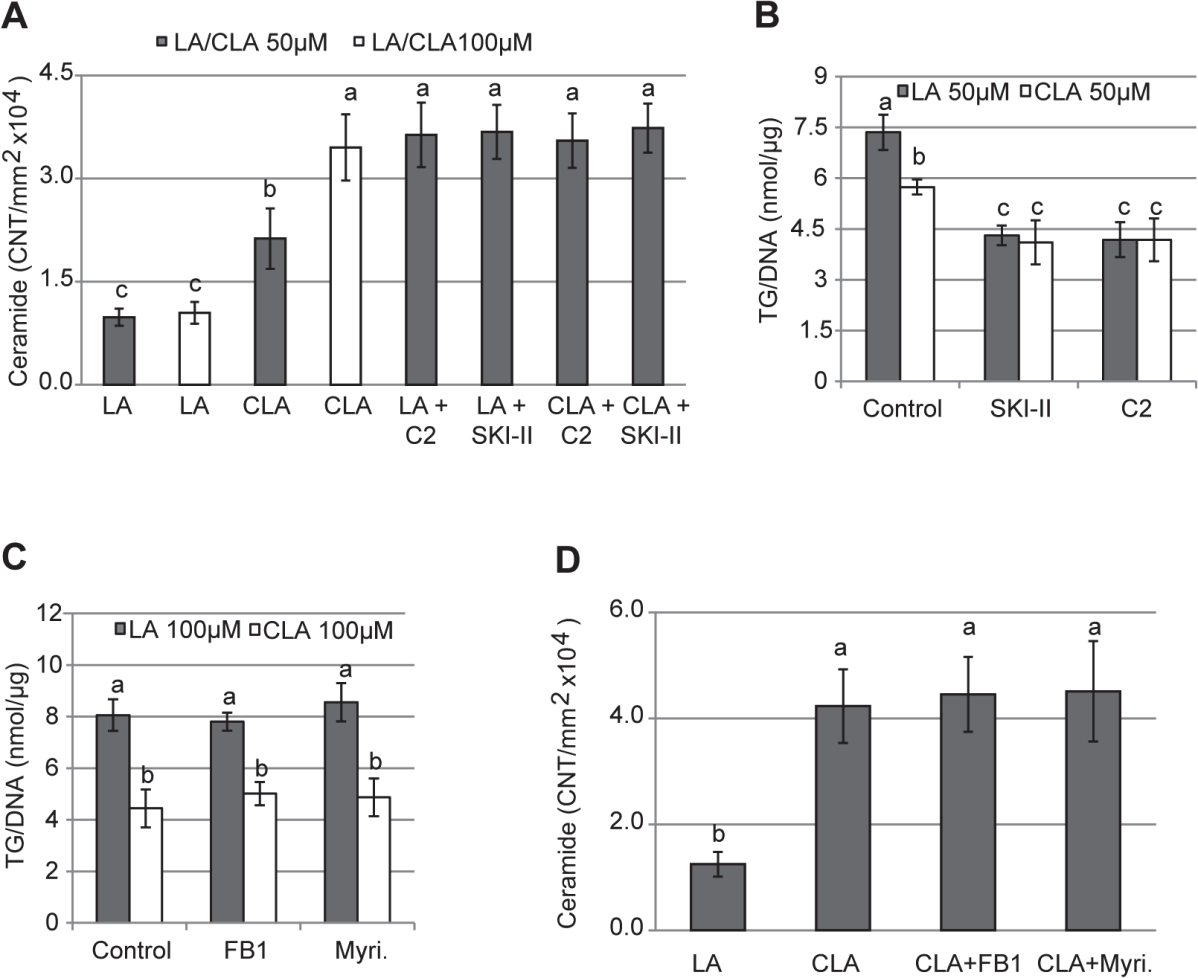
Ceramide and triacylglycerol levels in treated 3T3-L1 adipocytes. **(A)** Ceramide levels were measured with a monoclonal antibody assay in extracts from adipocytes 24 h after addition of 50 μM (gray bars) or 100 μM (white bars) LA or *t*10*c*12 CLA (CLA) with or without 30μM C2 ceramide (C2) or 10μg/ml of an inhibitor of sphingosine kinase (SKI-II), which is expected to increase ceramide levels. **(B)** Triacylglycerol (TG) levels 24 h after 3T3-L1 adipocytes were treated with 50 μM LA or *t*10*c*12 CLA (CLA), with or without 30 μM C2 ceramide or 10 μg/ml SKI-II. The control sample contained 0.2% DMSO, which produced the same values as a control sample containing 0.2% ethanol in separate experiments (data not shown). **(C)** Triacylglycerol levels 24 h after 3T3-L1 adipocytes were treated 100 μM LA or *t*10*c*12 CLA (CLA), with or without 20 μM fumonisin B1 (FB1) or 50 μM myriocin (Myri.). **(D)** Ceramide levels were detected and quantitated as in A for adipocytes treated for 24 h with 100 μM LA or *t*10*c*12 CLA, with or without 20 μM FB1 or 50 μM myriocin. Each bar represents the mean ± SEM (n = 3), and a representative experiment of at least three independent experiments is shown. Means not sharing a common letter differ, *p*≤0.05.

An immunoblot analysis of ceramide levels confirmed that they are elevated in adipocytes treated with 50 μM *t*10*c*12 CLA, and are present at higher levels if treated with 100 μM *t*10*c*12 CLA (Fig 2A). Thus, *t*10*c*12 CLA strongly increases ceramide/sphingolipid pools in adipocytes. To test the hypothesis that ceramides and/or sphingolipids are functionally involved in *t*10*c*12 CLA-mediated triacylglycerol loss in adipocytes, we tested two chemicals that enhance this pathway. C2 ceramide is a short chain ceramide that can mimic some ceramide responses [29]. SKI-II (sphingosine kinase inhibitor 2) is a chemical that inhibits sphingosine kinase 1, which normally depletes the sphingosine pool by producing sphingosine-1-phosphate (S1P, Fig 1). Addition of 30 μM C2 ceramide to adipocytes increased ceramide levels (Fig 2A). As the immunological assay used does not detect C2 ceramide (S2 Fig), this result indicates exogenous C2 ceramide increases endogenous ceramide levels). Thirty μM C2 ceramide resulted in strong reductions in triacylglycerol levels in the presence of LA, without further reductions in triacylglycerol levels in the presence of 50 μM *t*10*c*12 CLA (Fig 2B). Note this study uses 50 μM *t*10*c*12 CLA treatments when looking for effects that lower triacylglycerol levels. This is because 100 μM *t*10*c*12 CLA depletes triacylglycerol levels, making further reductions difficult to measure. Conversely, we use 100 μM *t*10*c*12 CLA when analyzing treatments that increase triacylglycerol levels, as having a lower triacylglycerol level as a reference point provides a larger range of measurable triacylglycerol increases.

Addition of SKI-II, which is expected to increase ceramide and sphingosine levels by inhibiting production of S1P from sphingosine (Fig 1), gives a similar result: ceramide levels increase in the presence of SKI-II and LA or *t*10*c*12 CLA (Fig 2A), and there is a strong loss of triacylglycerol levels adipocytes with these treatments (Fig 2B). The results that either C2 ceramide or SKI-II can mediate triacylglycerol loss in the absence of *t*10*c*12 CLA (Fig 2B) supports the premise that increased ceramide/sphingolipid levels are functionally involved in the response to *t*10*c*12 CLA. Surprisingly, there is not an additive effect on lowering triacylglycerol levels when these chemicals are combined with 50 μM *t*10*c*12 CLA.

A remaining question is whether ceramide/sphingolipid pathways are required for triacylglycerol loss in *t*10*c*12 CLA treated adipocytes. The combination of *de novo* biosynthesis and bioconversions between existing sphingolipid pools makes this a difficult question to answer. For example, adding fumonisin B1 or myriocin, both inhibitors of ceramide *de novo* biosynthesis (Fig 1), had little effect on the triacylglycerol levels in the presence of 100 μM *t*10*c*12 CLA (Fig 2C). Analysis of ceramide levels in adipocytes treated with 100 μM *t*10*c*12 CLA and these chemicals indicates that ceramide levels remained high in these cells despite the presence of these inhibitors (Fig 2D). This result suggests the ceramides are likely to be derived from both *de novo* biosynthesis and established pools within these adipocytes. Importantly, for interpreting subsequent experiments, these results indicate that these inhibitors did not have measurable cell toxicity or effects on triacylglycerol levels.

To gain more experimental control over inhibiting ceramide production, we developed short hairpin RNA (shRNA) knockdowns for serine palmitoyltransferase long chain subunit 1 (SPTLC) and S1P phosphatase (S1PP). Serine palmitoyltransferase (SPT1) catalyzes the initial step in ceramide biosynthesis and S1PP produces sphingosine from existing pools of S1P (Fig 1). Efficient shRNA knockdown of SPTLC protein levels was achieved as measured by immunoblot analysis (Table 2), but this knockdown increased triacylglycerol levels in *t*10*c*12 CLA treated adipocytes (Table 3), without decreasing ceramide levels (Table 3), making interpretation difficult. ShRNA knockdown of S1PP was also efficient as measured by immunoblot analysis (Table 2), and did not affect triacylglycerol levels in *t*10*c*12 CLA treated adipocytes (Table 3). Ceramide levels were still quite high when measured for the S1PP shRNA knockdowns in *t*10*c*12 CLA treated adipocytes (Table 3). Thus, shRNA knockdown of either of the SPTLC or S1PP enzymes was not sufficient to diminish ceramide levels.

**Table 2:** Protein levels of SPTLC and S1PP after shRNA knockdown.

Treatment	^1^SPTLC protein levels	S1PP protein levels
Control	1.2 ± 0.1^a^	1.2 ± 0.2^a^
shRNA non-specific	1.2 ± 0.3 ^a^	1.2 ± 0.1^a^
shRNA SPTLC or S1PP specific	0.5 ± 0.1 ^b^	0.5 ± 0.1 ^b^

^1^Immunoblot analysis of whole cell extracts isolated 24 h after 3T3-L1 adipocytes were treated with 100 nM of short hairpin RNA (shRNA) against non-target (shRNA non-specific), against Serine Palmitoyltransferase long chain subunit 1 (shRNA SPTLC), or against Sphingosine-1-Phosphate Phosphatase 1 (shRNA S1PP), or with transfection reagent only (Control).

A representative experiment of at least three independent experiments is shown. Means ± SEM (n = 3) not sharing a common letter differ, *p*≤0.05.

**Table 3:** Triacylglycerol and ceramide levels in shRNA treated cells.

Treatment	^1^Triacylglcerol levels nmol/μg		^2^Relative ceramide levels	
	100 μM LA	100 μM CLA	100 μM LA	100 μM CLA
shRNA non-specific	6.3 ± 0.4^a^	3.0 ± 0.5^c^	1.0 ± 0.1^b^	3.2 ± 0.3^a^
shRNA SPTLC	7.0 ± 0.6^a^	3.9 ± 0.2^bc^		3.4 ± 0.4^a^
shRNA S1PP	6.0 ± 0.6^a^	2.8 ± 0.5^c^		3.6 ± 0.2 ^a^

^1^Triacylglycerol or ^2^relative ceramide levels 24 h after cells were treated with 100 μM LA or *t*10*c*12 CLA and 100 nM of shRNA against non-target (non-specific), S1PP, or SPTLC sequences.

A representative experiment of at least three independent experiments is shown. Means ± SEM (n = 3) not sharing a common letter differ, *p*≤0.05.

**Fig 3:**
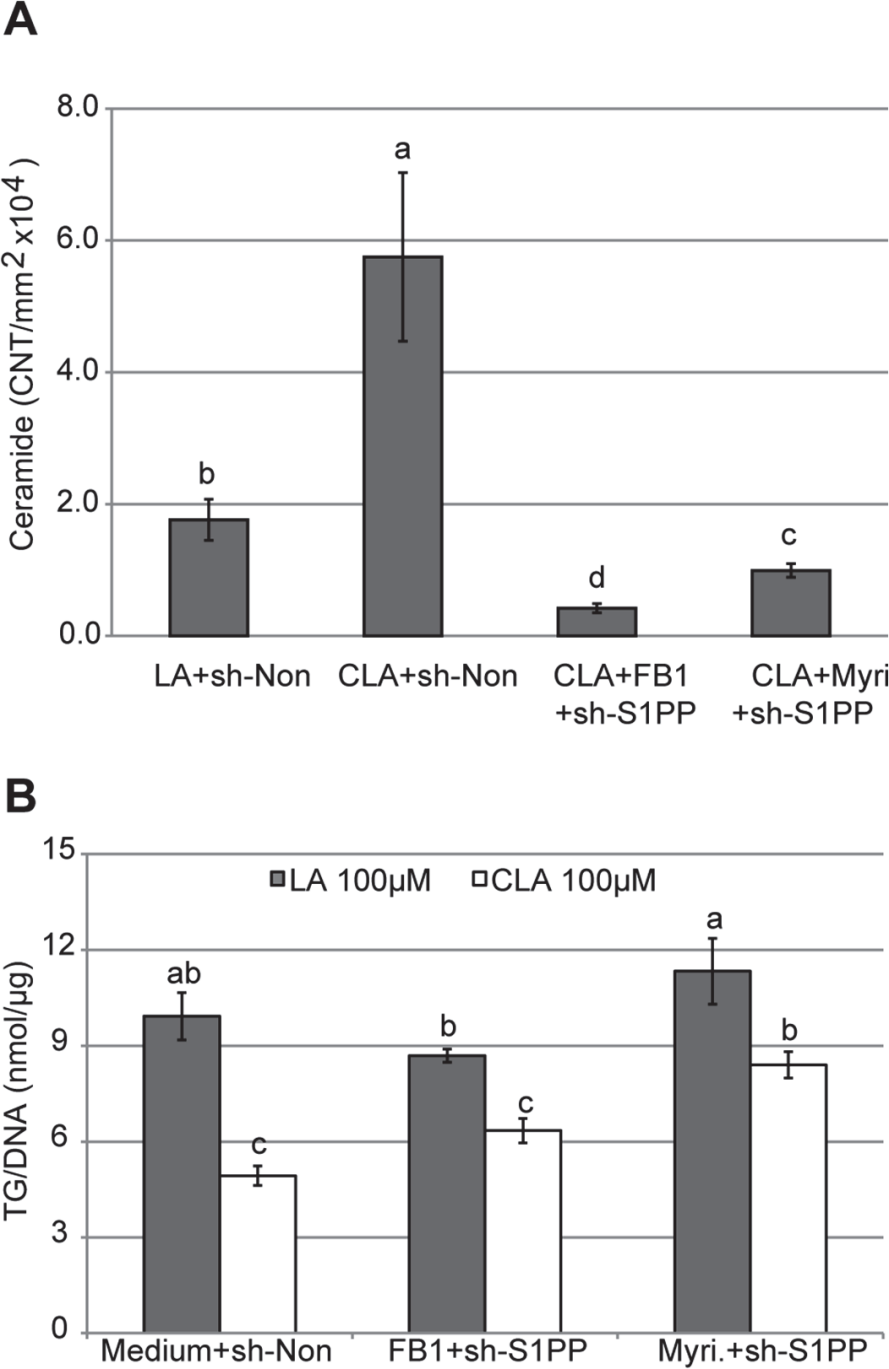
Inhibition of both biosynthetic entry points into ceramides affects triacylglycerol and ceramide levels. **(A)** Immunoblot analysis of ceramide levels 24 h after 3T3-L1 adipocytes were treated with 100 μM LA or *t*10*c*12 CLA in the presence of 100 nM shRNA against non-target (sh-Non) or S1PP (sh-S1PP), with or without 20 μM Fumonisin B1 (FB1) or 50 μM Myriocin (Myri). **(B)** Triacylglycerol (TG) levels after 3T3-L1 adipocytes were treated as in A. A representative experiment of at least three independent experiments is shown for each panel. Each bar represents the mean ± SEM (n = 3). Means not sharing a common letter differ, *p*≤0.05.

We next investigated whether ceramide levels could be reduced by concurrently inhibiting biosynthetic and S1P entry points into the pathway. A combination of myriocin, a SPT inhibitor, and shRNA against S1PP, is expected to inhibit both *de novo* biosynthesis of ceramides and their production from S1P (Fig 1). This combination was sufficient to strongly diminish ceramide levels in 100 μM *t*10*c*12 CLA treated adipocytes (Fig 3A). Further, this combination had higher triacylglycerol levels in the presence of 100 μM *t*10*c*12 CLA, relative to 100 μM *t*10*c*12 CLA alone, indicating it was effective in attenuating the ability of *t*10*c*12 CLA to cause triacylglycerol loss (Fig 3B).

A similar analysis was performed using a combination of shRNA against S1PP and fumonisin B1. Fumonisin B1 is an inhibitor of sphinganine N-acyltransferase (Fig 1), and thereby should inhibit ceramide biosynthesis. In combination with a shRNA knockdown S1PP, this should block both biosynthesis of ceramide and its generation from S1P (Fig 1). This combination was sufficient to strongly diminish ceramide levels in 100 μM *t*10*c*12 CLA treated adipocytes (Fig 3A). These fumonisin B1 and S1PP shRNA/(LA or *t*10*c*12 CLA) combination treatments had a higher ratio of triacylglycerol^CLA^/ triacylglycerol^LA^ than the control lacking fumonisin (Fig 3B), in agreement with the similar myriocin experiment described above (Fig 3B). However, there was a tendency for the fumonisin B1 and S1PP shRNA/LA combination treatment to have lower triacylglycerol levels than the non-specific shRNA and LA combination control lacking fumonisin (Fig 3B). As this suggests some possible off-target effects with fumonisin B1, our analyses rely primarily on the results with myriocin and S1PP shRNA to support the hypothesis that ceramides mediate much of the triacylglycerol lowering effects in *t*10*c*12 CLA treated adipocytes.

### Ceramide signaling is downstream of AMPK and can increase AMPK activity

AMPK is a key component of responses in *t*10*c*12 CLA treated adipocytes, as its inhibition by compound C abolishes the effects of *t*10*c*12 CLA [6]. As demonstrated above (Fig 2B), either C2 or SKI-II is sufficient to mediate triacylglycerol loss in adipocytes in the absence of *t*10*c*12 CLA, raising the question of whether ceramide-mediated triacylglycerol loss requires activated AMPK. To answer this, adipocytes were treated with C2 or SKI-II in the presence or absence of 10 μM of AMPK inhibitor compound C (Table 4). For comparison, the ability of compound C to interfere with triacylglycerol loss in the presence of 100 μM *t*10*c*12 CLA is also shown. A considerable reduction in triacylglycerol levels occurs in the presence of C2 or SKI-II, but this response is not affected by the presence of compound C (Table 4). Therefore, we conclude that C2 and SKI-II act downstream or independently of AMPK to mediate triacylglycerol loss in adipocytes.

**Table 4:** Triacylglycerol levels in 3T3-L1 adipocytes treated with C2-ceramide, SKI-II, or *t*10*c*12 CLA, with or without compound C.

	^1^Triacylglcerol levels nmol/μg	
Treatment	No Compound C	10 μM Compound C
Control	6.3 ± 0.7^a^	6.8 ± 0.5^a^
30 μM C2 ceramide	3.7 ± 0.5^b^	3.6 ± 0.4^b^
10 μg/ml SKI-II	3.4 ± 0.3^b^	3.5 ± 0.5^b^
100 μM *t*10*c*12 CLA	3.6 ± 0.3^b^	7.1 ± 0.4^a^

^1^The means ± SEM (n = 3) of a representative experiment of at least three independent experiments is shown. Means not sharing a common letter differ, *p*≤0.05.

We also measured the activity of AMPK in the presence of these compounds. The active form of AMPK can be detected with an antibody specific for its phosphorylated active state at AMPKα pThr172 [30]. 50 μM *t*10*c*12 CLA activates p-AMPK, relative to p-AMPK levels in LA treated adipocytes (Table 5). Surprisingly, both C2 and SKI-II activated AMPK in 50 μM LA or *t*10*c*12 CLA to equivalent p-AMPK levels (Table 5). Thus, although AMPK is not required for triacylglycerol loss in C2 or SKI-II treated adipocytes as indicated by the similar losses with or without the presence of compound C, these compounds activate AMPK.

**Table 5:** C2 ceramide, SKI-II, or *t*10*c*12 CLA activate AMPK.

	Relative p-AMPK levels		
Fatty Acid	None	30 μM C2 ceramide	10 μg/ml SKI-II
50 μM LA	0.1 ± 0.04^b^	1.0 ± 0.3^a^	0.9 ± 0.3^a^
50 μM CLA	1.0 ± 0.2^a^	0.9 ± 0.1^a^	1.1 ± 0.1^a^

Immunoblot analyses determined the relative values of the ratio of activated AMPK (phospho-AMPKα: pThr172) to total AMPK from cytoplasmic proteins isolated 12 h after addition of 50 μM LA or t10c12 CLA, with or without 30 μM C2-ceramide or 10 μg/ml SKI-II. Control solvent vehicle indicated by ‘None’. The means ± SEM (n = 3) of a representative experiment of at least three independent experiments is shown. Means not sharing a common letter differ, p≤0.05.

### C2 and SKI-II activate NF-κB independently of AMPK

Activation of NF-κB has an important functional role in mediating triacylglycerol loss in *t*10*c*12 CLA treated adipocytes [3,14]. As an additional comparison between the response of adipocytes to *t*10*c*12 CLA and their response to C2 or SKI-II, we examined the amount of active NF-κB present in the nucleus. The ability of 100 μM *t*10*c*12 CLA treatment to activate NF-κB is partially dependent on AMPK as compound C attenuates this response (Table 6). Treatment with either C2 or SKI-II activates NF-κB in adipocytes in the presence of either LA or *t*10*c*12 CLA (Table 7). NF-κB activation occurs equally in C2 or SKI-II treated adipocytes whether compound C is present or absent (Table 8). These results indicate that C2 or SKI-II act downstream or independently of AMPK to activate NF-κB.

**Table 6:** Relative NF-κB nuclear levels in adipocytes treated with LA or *t*10*c*12 CLA, with or without Compound C.

	Relative NF-κB nuclear levels	
Treatment	No Compound C	10 μM Compound C
100 μM LA	1.0 ± 0.2^c^	1.3 ± 0.2^c^
100 μM CLA	4.0 ± 0.7 ^a^	3.1 ± 0.5^b^

Nuclear extracts were analyzed by immunoblot for NF-κB and β-actin, and the relative levels of NF-κB/β-actin are displayed for the indicated treatments. The means ± SEM (n = 3) of a representative experiment of at least three independent experiments is shown. Means not sharing a common letter differ, p≤0.05.

**Table 7:** Relative NF-κB nuclear levels in adipocytes treated with LA or *t*10*c*12 CLA, with or without C2-Ceramide or SKI-II.

	Relative NF-κB nuclear levels		
Fatty Acid	None	30 μM C2 ceramide	10 μg/ml SKI-II
50 μM LA	1.0 ± 0.1^b^	4.0 ± 0.3^a^	4.0 ± 0.6^a^
50 μM CLA	3.6 ± 0.6^a^	4.2 ± 0.6^a^	4.0 ± 0.4^a^

Nuclear extracts were analyzed by immunoblot for NF-κB and β-actin, and the relative levels of NF-κB/β-actin are displayed for the indicated treatments. Control solvent vehicle indicated by ‘None’. The means ± SEM (n = 3) of a representative experiment of at least three independent experiments is shown. Means not sharing a common letter differ, p≤0.05.

**Table 8:** Relative NF-κB nuclear levels in adipocytes treated with C2-Ceramide or SKI-II, with or without Compound C.

	Relative NF-κB nuclear levels		
Compound C	None	30 μM C2 ceramide	10 μg/ml SKI-II
None	1.2 ± 0.4^b^	3.4 ± 0.5^a^	3.7 ± 0.7^a^
10 μM	1.0 ± 0.2 ^b^	3.6 ± 0.6^a^	3.6 ± 0.5^a^

Nuclear extracts were analyzed by immunoblot for NF-κB and β-actin, and the relative levels of NF-κB/β-actin are displayed for the indicated treatments. Control solvent vehicle indicated by ‘None’. The means ± SEM (n = 3) of a representative experiment of at least three independent experiments is shown. Means not sharing a common letter differ, p≤0.05.

### Loss of cell adherence in *t*10*c*12 CLA treated adipocytes is a ceramide-dependent response

Measurement of the amounts of cellular DNA per well of treated adipocytes supports a role for *t*10*c*12 CLA in causing a loss of cell adherence. *T*10*c*12 CLA treated adipocytes have less total DNA (Table 6) and more non-attached cells when examined microscopically (data not shown). These detached cells are removed when removing the media and washing the adherent cells prior to harvesting the adherent cells for analysis. Addition of C2 ceramide or SKI-II to LA treated adipocytes caused cell loss similar to those treated with 100 μM *t*10*c*12 CLA as indicated by the lower amounts of total cellular DNA (Table 9). Combinations of C2 ceramide or SKI-II with 50 μM LA produced about the same amount of cell loss as treatment with 100 μM *t*10*c*12 CLA. Combining either of these drugs with 50 μM *t*10*c*12 CLA did not significantly increase the amount of cell loss (Table 9). Inhibition of the ceramide pool levels in *t*10*c*12 CLA treated adipocytes by concurrent inhibition of ceramide biosynthesis and entry from S1P by combinations of myriocin or FB1 and knockdown of SIPP by shRNA attenuated cell loss (Table 10). These results suggest that *t*10*c*12 CLA-mediated loss of cell adherence in adipocytes is mediated in part or predominantly by ceramides.

**Table 9:** DNA amounts isolated from adherent cells after various chemical treatments.

Treatments	% DNA		
	None	C2 ceramide	SKI-II
50 μM LA	100 ± 2^a^	87 ± 3^bc^	86 ± 3^c^
50 μM CLA	93 ± 2^b^	83 ± 3^c^	83 ± 4^c^
100 μM LA	101 ± 4^a^		
100 μM CLA	85 ± 2^c^		

3T3-L1 adipocytes were assayed for DNA amounts per well as an indicator of cell attachment after 24 h of the indicated treatments. DNA amounts after 50 or 100 μM LA or *t*10*c*12 CLA treatments, with or without 30 μM C2-ceramide or 10 μg/ml SKI-II, as a percentage of the sample treated with 50 μM LA. A representative experiment of at least three independent experiments is shown for each set of treatments. Control solvent vehicle indicated by ‘None’. The mean ± SEM (n = 3) of three replicates is shown. Means not sharing a common letter differ, *p*≤0.05.

**Table 10:** DNA amounts isolated from adherent cells after adding LA or *t10c12* CLA and various shRNAs.

Treatments	% DNA		
	sh-Non-target	sh-S1PP + Myriocin	sh-S1PP + FB1
100 μM LA^1^	100 ± 3^a^	99 ± 4^ab^	99 ± 2^ab^
100 μM CLA	86 ± 5^d^	92 ± 2^c^	94 ± 3^bc^

3T3-L1 adipocytes were assayed for DNA amounts per well as an indicator of cell attachment after 24 h of the indicated treatments. A representative experiment of at least three independent experiments is shown for each set of treatments. The mean ± SEM (n = 3) of three replicates is shown. Means not sharing a common letter differ, *p*≤0.05.

^1^DNA amounts after 100 μM LA or *t*10*c*12 CLA treatments, with non-target shRNA (sh-Non-target) or shRNA targeting S1PP (sh-S1PP) and either 50 μM myriocin or 20 μM Fumonisin B1 (FB1).

## Discussion

Our metabolite profiling analysis of adipocytes treated with *t*10*c*12 CLA indicated there were increased sphinganine and sphingosine levels in the ceramide pathway after 12 h, but not at the earlier 0.5 h time point. The metabolite profiling did not analyze ceramides, but our antibody-based detection method indicated ceramide levels were increased as well. The experiments blocking both entry points into the ceramide pathway attenuated ceramide levels as measured by the monoclonal antibody assay. As single block experiments did not block ceramide levels according to this assay and thereby serve as controls for the double block experiments, these results indicate the monoclonal antibody against ceramides demonstrates preferential specificity for ceramides over sphingomyelin, cholesterol or other phospholipids. The agreement between the mass spectrometry measurements of increased sphinganine and sphingosine levels and the monoclonal assays of ceramides strongly supports the conclusion that *t*10*c*12 CLA treatments increase sphingolipid levels in adipocytes.

An earlier report on the effects of *t*10*c*12 CLA in adipocytes observed that the rate of ceramide biosynthesis was attenuated when measured from radiolabeled acetate or pyruvate [31]. This is consistent with the reduced rate of *de novo* fatty acid biosynthesis observed in *t*10*c*12 CLA treated adipocytes [7,31]. However, the abundant pools of pre-existing fatty acids and sphingolipids in adipocytes should allow for ceramide synthesis and salvage from existing molecules even when *de novo* fatty acid biosynthesis from two-carbon units is attenuated. Therefore, as total ceramide levels were not directly measured in this earlier report, it does not contradict our findings of increased ceramide levels in *t*10*c*12 CLA treated adipocytes.

Our inhibitor studies indicated inhibiting ceramide levels required blocking both the *de novo* ‘sphinganine’ entry point as well as the salvage ‘sphingosine’ entry point (see Fig 1). As both the sphinganine and sphingosine levels were elevated *in vivo*, this suggests increased flux from the unidirectional sphinganine to ceramide steps. Alternatively, ceramide levels could be increased via both of the sphinganine and sphingosine entry points. Our observation that we needed to block both of these entry points in our double block experiments (myriocin and S1PP shRNA) suggests both entry points are active in producing ceramides in adipocytes. This requirement to block both entry points into ceramide production to block the effects of *t*10*c*12 CLA suggests increased flux through the ceramide pathway to produce the more complex sphingomyelins, cerebrosides, and/or gangliosides is not functionally important as a single block of this biosynthetic pathway should have been sufficient for blocking their production.

Effectively blocking ceramide levels attenuated the effects of *t*10*c*12 CLA, supporting a functional role for elevated ceramides and/or sphinganine and/or sphingosine in lowering triacylglycerol levels. A functional role is also supported by our result that adding C2 ceramide or the sphingosine kinase inhibitor SKI-II increased endogenous ceramide levels and lowered triacylglycerol levels. The combination of increased ceramide levels in cells, an attenuation of the triacylglycerol lowering response when ceramide levels are blocked, and a triacylglycerol lowering response when ceramides are increased, all support the conclusion that molecules in the ceramide pathway are functionally important in the triacylglycerol lowering response in *t*10*c*12 CLA-treated adipocytes.

We discuss some of the known responses in adipocytes treated with *t*10*c*12 CLA [5] with regards to possible roles of ceramide or ceramide-related molecules in these responses. *T*10*c*12 CLA affects a number of biological processes including: increased energy expenditure and fatty acid oxidation, repression of fatty acid biosynthesis, and activation of AMPK [6,7,31,32]; increased NF-κB and inflammation [3,14]; and apoptosis in adipocytes and adipose tissues [11,33].

### Energy metabolism and ceramide activation of AMPK

Activated AMPK is required for triacylglycerol loss in *t*10*c*12 CLA-treated adipocytes [6,14]. Activated AMPK increases fatty acid oxidation and energy production while decreasing fatty acid biosynthesis in most cells [34], including *t*10*c*12 CLA-treated adipocytes [6,7]. Activation of AMPK occurs as early as 0.5 h after addition of *t*10*c*12 CLA [6]. In contrast, sphinganine and sphingosine levels are not significantly elevated at this time (Table 1), suggesting the elevation of these occurs after AMPK activation. Addition of C2 ceramide activated AMPK in adipocytes as described here or in an INS-1 pancreatic beta cell line [35]. Sphingosine, which was elevated 3.6 fold in our *t*10*c*12 CLA-treated adipocytes, can be phosphorylated by sphingosine kinase to produce S1P, which activates AMPK in hepatocytes [36]. FTY720, an analog of S1P, can stimulate AMPK activity in adipocytes and is associated with an anti-obesity effect in obese mice [37]. Activated AMPK increases fatty acid oxidation in mitochondria by a number of mechanisms [34]. Additionally, ceramides occur in mitochondria and can affect mitochondrial membrane permeability, respiratory chain function, oxidative phosphorylation, and apoptosis [17,38]. Increased mitochondrial ceramide levels may account in part for some of the increased fatty acid oxidation occurring in *t*10*c*12 CLA-treated adipocytes [6,31].

### Ceramides activate NF-κB in adipocytes

Increased NF-κB activity is observed in *t*10*c*12 CLA treated adipocytes and this increased activity is necessary for the triacylglycerol loss response in adipocytes [3]. NF-κB activity is associated with an inflammatory response, including prostaglandin biosynthesis, which is also required for triacylglycerol loss in *t*10*c*12 CLA treated adipocytes [12,14]. Sphingolipid metabolites, including ceramides, sphingosine, and S1P activate NF-κB in a variety of cells including fibroblasts [39], bronchial epithelium cells [40], endothelial cells [41], mouse adipocytes [42], and intestinal cells [43]. Our results demonstrate that *t*10*c*12 CLA, C2 ceramide, or SKI-II increased ceramide/sphingolipid levels and activated NF-κB in adipocytes. Taken together with the observation of increased ceramide, sphinganine, and sphingosine levels, these data suggest that increased sphingolipid metabolites could be fully or partially responsible for NF-κB activation in the triacylglycerol loss response of adipocytes treated with *t*10*c*12 CLA.

### Loss of cell adherence and apoptosis in adipocytes and adipose tissues

*T*10*c*12 CLA mediates reductions in mouse white adipose tissue cell numbers in part by apoptosis [11,33]. Ceramides stimulate apoptosis in adipocytes *in vitro* [44,45]. In our current studies, we did not specifically measure apoptosis, but *t*10*c*12 CLA or increased ceramide levels caused about 15% of the adipocytes to become non-adherent, presumably due to their becoming apoptotic. Concurrent inhibition of ceramide *de novo* biosynthesis and entry from S1P attenuated *t*10*c*12 CLA-mediated loss of cell adherence. Our results suggest the ceramide/sphingolipid pathway is a major signaling pathway in *t*10*c*12 CLA-mediated loss of cell adherence. Note that as our triacylglycerol measurements are per μg of cellular DNA, this measurement is not affected by the loss of the non-adherent cells. However, apoptosis and loss of adipocytes in mouse white adipose tissues significantly contributes to overall reductions in the mass of these tissues [11,33]. Our findings of increased sphinganine, sphingosine, and ceramide levels in *t*10*c*12 CLA treated adipocytes provide a biochemical explanation for the apoptosis and loss of adipocytes observed in white adipose tissues of mice fed *t*10*c*12 CLA [11,33].

### Effect of sphingolipid metabolites in whole animal studies

The triacylglycerol lowering effects of sphingolipid metabolites in adipocytes *in vitro* is in agreement with a study utilizing FTY720, an analog of S1P, that lowers triacylglycerol levels in adipocytes *in vitro* and has an anti-obesity effect in obese mice [37]. Mice fed *t*10*c*12 CLA diets exhibit rapid loss of white adipose tissue [1,2,11]. A hypothesis that similar mechanisms occur in animals and *in vitro* is supported by our earlier studies demonstrating the whole genome transcriptional responses of white adipose tissue of mice fed *t*10*c*12 CLA and of 3T3-L1 adipocytes treated with *t*10*c*12 CLA are very similar when measured by microarrays [9]. If a hypothesis that the mechanisms are similar is correct, then a corollary is that sphingolipid metabolites are an important component of the fat loss response of white adipose tissue in mice fed *t*10*c*12 CLA. However, a general conclusion that treatments that increase sphingolipid metabolites cause fat loss in animals is opposed by an experiment in mice where inhibition of ceramide *de novo* biosynthesis with myriocin attenuated obesity symptoms, facilitating weight reduction, better energy metabolism, and improved insulin sensitivity [18]. These contradictory results from animal trials suggest a simple generalization of the effects of perturbing the complex ceramide/sphingolipid pathway in whole animal studies may not be possible. However, for *in vitro* cultures, where adipocytes are often 60 to 90 percent of the cells, ceramides and sphingolipid metabolites are associated with lowering triacylglycerol levels and/or apoptosis [19,37,46,47].

Our results suggest that increased sphingolipid metabolites should be added to the list of mechanisms implicated in lowering triacylglycerol in adipocytes treated with *t*10*c*12 CLA, although the mechanism by which *t*10*c*12 CLA activates sphingolipid biosynthesis remains unknown. Increased sphingolipid metabolites alter the physical properties of cell membranes where they can create lipid rafts, alter membrane fluidity, and interact with key membrane proteins to activate ceramide/sphingolipid signaling pathways [48].

## Supporting Information

S1 Dataset Metabolite levels of 261 metabolites in LA and *t*10*c*12 CLA treated adipocytes. 3T3-L1 adipocytes were treated with 100 μM LA (LA) or *t*10*c*12 CLA for 0.5 or 12 h and harvested for analysis of 261 metabolites (Materials and Methods).
(XLS)

S1 Fig Comparison of methods of preparing cellular triacylglycerol for enzymatic assay. A comparison of our ‘whole-cell sonication’ method with an isopropanol-hexane extraction method was performed. The whole cell sonication method is described in the Materials and Methods section. The isopropanol-hexane extraction method was performed as described [28]. A series of different amounts of adipocytes were used in the two methods and the triacylglycerol levels measured as described in Materials and Methods. Both assays were linear over the range of cells measured (Note the range of triacylglycerol levels typically measured is close to the mid-point of the range shown). The SEM of the isopropanol-hexane extraction method is larger than the sonication method, while the linear equation for the sonication method has a smaller slope. These results indicate the sonication method is more reproducible and slightly underestimates differences in triacylglycerol levels. A representative experiment from two independent experiments is shown and each bar represents the mean ± SEM (n = 3).
(TIF)

S2 Fig The monoclonal antibody recognizing ceramides does not detect C2 ceramide. 10 and 20 nmol of C2 ceramide were spotted onto PVDF membrane as well as lipid soluble extracts from adipocytes treated with 50 μM or 100 μM *t*10*c*12 CLA (CLA) and analyzed by immunoblot analysis with a monoclonal antibody that detects ceramides. Complete recovery of the 60 nmol of C2 ceramide used per well at the standard 30 μM concentration would result in 1.2 nmol of C2 per spot as only 2% of the sample is spotted. The 10 and 20 nmol amounts are approximately 8 fold and 17 fold more than the maximum C2 levels possible in the C2 treated samples. Therefore, we conclude that the monoclonal antibody used in the immunoblot assay does not detect C2 ceramide at the levels added to the cells.
(TIF)
