# Supplementary figures and images for: Sphingolipids Are Required for Efficient Triacylglycerol Loss in Conjugated Linoleic Acid Treated Adipocytes
Source: PLoS One. 2015 Apr 23;10(4):e0119005. doi: 10.1371/journal.pone.0119005 (PMC4407960; doi:10.1371/journal.pone.0119005)

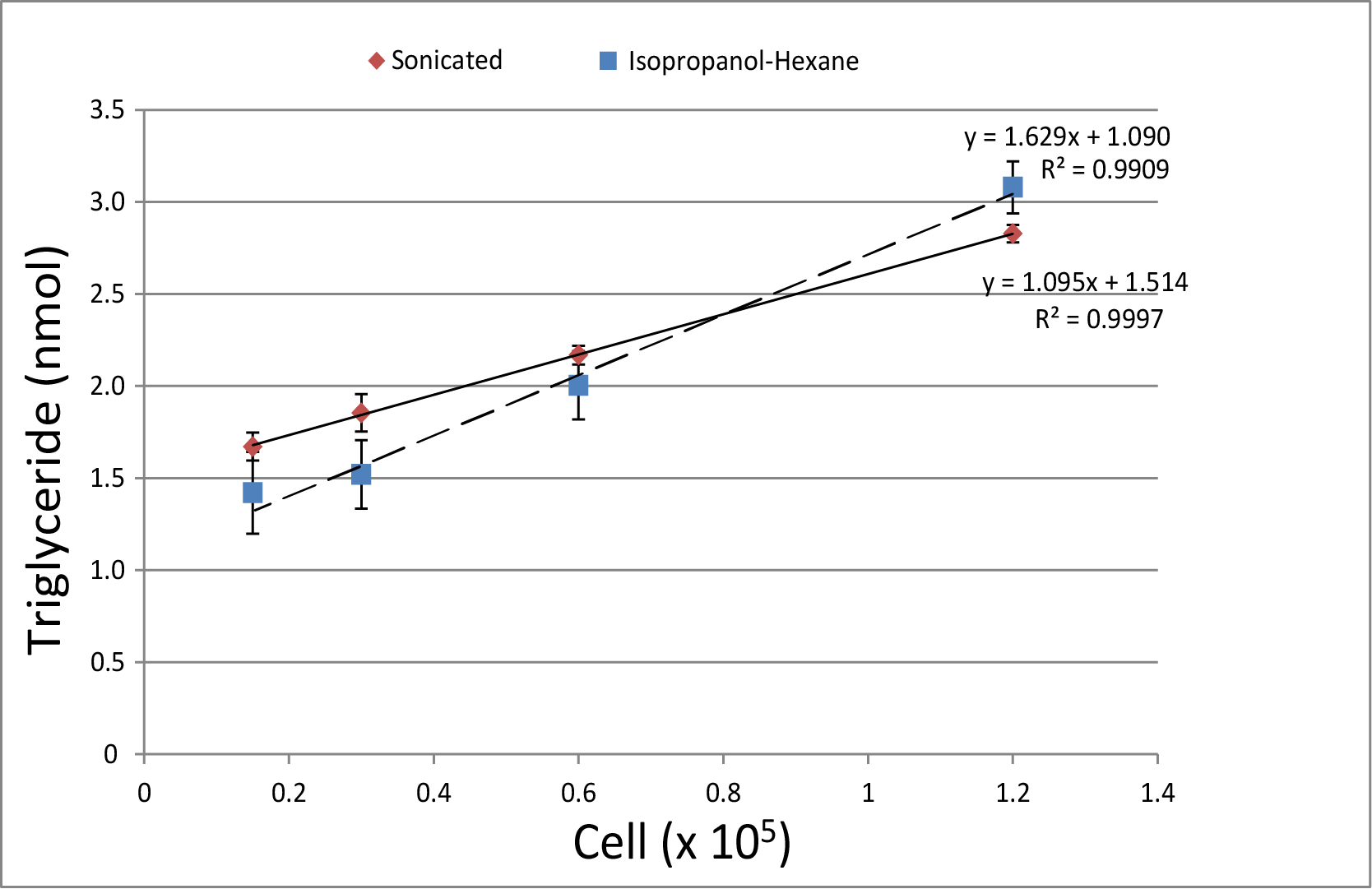

Supplement: S1 Fig — A comparison of our ‘whole-cell sonication’ method with an isopropanol-hexane extraction method was performed. The whole cell sonication method is described in the Materials and Methods section. The isopropanol-hexane extraction method was performed as described [28]. A series of different amounts of adipocytes were used in the two methods and the triacylglycerol levels measured as described in Materials and Methods. Both assays were linear over the range of cells measured (Note the range of triacylglycerol levels typically measured is close to the mid-point of the range shown). The SEM of the isopropanol-hexane extraction method is larger than the sonication method, while the linear equation for the sonication method has a smaller slope. These results indicate the sonication method is more reproducible and slightly underestimates differences in triacylglycerol levels. A representative experiment from two independent experiments is shown and each bar represents the mean ± SEM (n = 3). (TIF) [file pone.0119005.s002.tif]

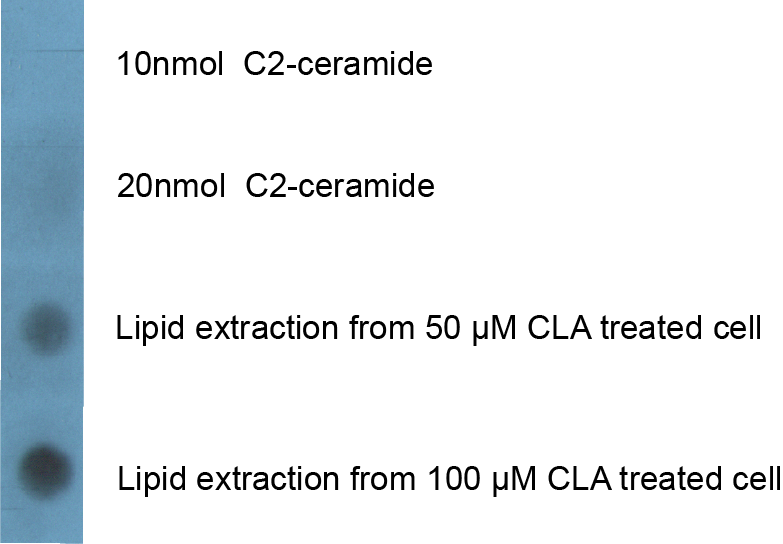

Supplement: S2 Fig — 10 and 20 nmol of C2 ceramide were spotted onto PVDF membrane as well as lipid soluble extracts from adipocytes treated with 50 μM or 100 μM t10c12 CLA (CLA) and analyzed by immunoblot analysis with a monoclonal antibody that detects ceramides. Complete recovery of the 60 nmol of C2 ceramide used per well at the standard 30 μM concentration would result in 1.2 nmol of C2 per spot as only 2% of the sample is spotted. The 10 and 20 nmol amounts are approximately 8 fold and 17 fold more than the maximum C2 levels possible in the C2 treated samples. Therefore, we conclude that the monoclonal antibody used in the immunoblot assay does not detect C2 ceramide at the levels added to the cells. (TIF) [file pone.0119005.s003.tif]
